# SREBP2 inhibitor betulin sensitizes hepatocellular carcinoma to lenvatinib by inhibiting the mTOR/IL-1β pathway: SREBP2 Inhibitor sensitizes HCC to lenvatinib

**DOI:** 10.3724/abbs.2023122

**Published:** 2023-07-11

**Authors:** Minghao Fan, Zhenmei Chen, Weiqing Shao, Yiran Chen, Zhifei Lin, Chenhe Yi, Yitong Li, Lu Lu, Yu Zhou, Jing Lin

**Affiliations:** 1 Department of General Surgery Huashan Hospital Fudan University Shanghai 200040 China; 2 Department of Infectious Diseases the Third Afflicted Hospital of Wenzhou Medical University Wenzhou 325200 China

**Keywords:** hepatocellular carcinoma, SREBP2, lenvatinib, betulin, cholesterol, interleukin-1β

## Abstract

Lenvatinib has become the first-line therapy in advanced hepatocellular carcinoma (HCC), but its efficacy is still limited because of the inevitable development of resistance. It has been reported that cellular cholesterol levels are associated with tyrosine kinase inhibitor (TKI) efficacy. Here, we show that betulin, a sterol regulatory element-binding protein 2 (SREBP2) inhibitor, markedly enhances the anti-tumor effect of lenvatinib in HCC both *in vitro* and *in vivo*. Our results also show that the combination treatment of lenvatinib and betulin synergistically inhibits the proliferation and clonogenicity of HCC cells. The mRNA and protein expressions of IL-1β are markedly decreased in HCC cells treated with betulin, while the sensitivity of HCC cells to lenvatinib is enhanced. Moreover, we find that the knockdown of *IL-1β* also enhances the efficacy of lenvatinib, and recombinant IL-1β protein rescues cell viability, which is reduced by lenvatinib in HCC cells. Further mechanistic studies indicate that betulin decreases the level of IL-1β in HCC cells by inhibiting the mTOR signaling pathway. Finally, the growth of the tumors in xenograft mouse models subjected to combination treatment is significantly suppressed. In summary, our study reveals that the SREBP2 inhibitor betulin sensitizes hepatocellular carcinoma to lenvatinib by inhibiting the mTOR/IL-1β pathway, which may be a promising therapeutic strategy for patients with HCC.

## Introduction

Hepatocellular carcinoma (HCC) is the second leading cause of cancer-related death worldwide [ 1 , 2]. Targeted therapy has been reported to play an important role in systemic therapy for advanced HCC. Lenvatinib, a tyrosine kinase inhibitor, has become a first-line treatment option in advanced HCC. However, the efficacy of lenvatinib in patients with HCC is limited due to the inevitable development of drug resistance [ 3, 4]. Therefore, it is imperative to find a safe and effective combination treatment strategy for anti-HCC therapy.

Many previous studies have shown that aberrant accumulation of cholesterol is a common feature of tumor cells and that the level of intracellular cholesterol is associated with the efficacy of TKIs [ 5‒ 7]. Notably, our previous work revealed that a liver X receptor (LXR) agonist could enhance the anti-tumor activity of sorafenib in HCC cells by promoting cholesterol efflux [8]. Sterol regulatory element-binding protein 2 (SREBP2) is a secondary transmembrane protein located in the endoplasmic reticulum that plays a central role in regulating cholesterol homeostasis. Inhibition of SREBP2 has shown a broad-spectrum anti-tumor effect in many types of cancers by reducing intracellular cholesterol level [ 9‒ 11]. Thus, we hypothesized that SREBP2 inhibitors might enhance the sensitivity of HCC cells to TKIs. This assumption was confirmed in our study.

Previous studies have shown that intracellular cholesterol level is associated with the sensitivity of HCC cells to TKIs. However, the mechanism of how reduced cellular cholesterol enhances sensitivity requires further investigation. As a master regulator of cell growth, proliferation and survival, mammalian rapamycin target protein (mTOR) was reported to be inhibited when the level of intracellular cholesterol was decreased. In addition, increased cholesterol level promotes the release of a series of inflammatory factors, such as interleukin-6 (IL-6), IL-1β, and tumor necrosis factor-α (TNF-α) [ 11‒ 13]. Many studies have shown that SREBP2 expression is closely related to the mTOR pathway. As reported previously, SREBP activity is regulated by mTOR, and SREBP2 promotes proliferation downstream of the mTOR pathway [ 14 , 15]. Therefore, it is reasonable to assume that the mTOR signaling pathway and inflammatory factors may be associated with the betulin-mediated enhancement of the anti-tumor effect of lenvatinib.

In the present study, we revealed that the SREBP2 inhibitor betulin enhances the efficacy of lenvatinib in HCC both *in vitro* and *in vivo*. Further mechanistic studies indicated that betulin sensitizes HCC to lenvatinib by inhibiting the mTOR/IL-1β pathway. Our results highlight that combining an SREBP2 inhibitor with lenvatinib is potentially an effective therapeutic strategy in HCC.

## Materials and Methods

### Cell culture

The human HCC cell lines MHCC97H, HUH7, PLC/PRF/5, and HepG2 were obtained from Liver Cancer Institute of Fudan University (Shanghai, China). All cell lines were cultured in DMEM (Gibco, Carlsbad, USA) supplemented with 10% FBS at 37°C with 5% CO _2_.

### Clinical specimens

HCC tissues were collected from patients who underwent hepatectomy for HCC at the Department of General Surgery, Huashan Hospital, Fudan University (Shanghai, China). Informed consents were obtained from all participants before enrolment in this study. None of the patients received any preoperative cancer treatment. Ethical permission was obtained from the Ethics Committee of Huashan Hospital, Fudan University.

### Cell growth assay

For the cell proliferation assay, cells were seeded in 96-well plates at 5000 cells per well, and incubated in the presence of betulin or rapamycin with lenvatinib (Selleck Chemicals, Houston, USA) at the indicated concentrations. The Cell Counting Kit-8 (CCK-8; Dojindo Laboratories, Tokyo, Japan) was used to evaluate cell proliferation daily over a 72-h time course. Cells treated with 0.1% DMSO served as the vehicle control. The absorbance at a wavelength of 450 nm was measured to estimate the viable cells in each well.

### Immunohistochemistry (IHC)

Tissue samples were prepared and preserved through paraffin embedding and then dewaxed and blocked in a hydrogen peroxide/methanol solution. Antigen retrieval was performed using sodium citrate (pH 6.0) for 2×20 min at 80°C. After that, the sections were incubated with anti-SREBP2 antibody (Abcam, Cambridge, UK) and anti-IL-1β antibody (Proteintech, Chicago, USA) overnight at 4°C, followed by incubation with HRP-conjugated anti-rabbit IgG secondary antibody (Abcam) at 37°C for 30 min. The sections were stained with DAB and counterstained with hematoxylin, dehydrated in ethanol, mounted in dimethylbenzene, and placed under a coverslip. Analysis of IHC images was performed using ImageJ (NIH, Bethesda, USA).

### Western blot analysis

Cells were lysed in RIPA buffer, and then lysates were centrifuged at 13,400 *g* for 10 min and quantified using a BCA protein assay kit (Beyotime, Shanghai, China). Samples were separated on 8%‒12% SDS-PAGE gels and transferred to PVDF membranes (IPVH00010; Millipore, Billerica, USA). The membrane was blocked for 1 h at room temperature with 5% milk solution in TBS-Tween [50 mM Tris (pH 8.0), containing 150 mM NaCl and 0.1% Tween 20] before incubation with primary antibodies, including anti-SREBP2 antibody (Abcam), anti-IL-1β antibody (Proteintech), anti-mTOR antibody, and anti-pmTOR antibody (Cell Signaling Technology, Danvers, USA) at 4°C overnight. Subsequently, HRP-conjugated secondary antibody anti-rabbit IgG was added, followed by incubation at room temperature for 1 h. Western blots were visualized using chemiluminescence reagents (Sigma, St Louis, USA).

### Quantitative real-time PCR

Total RNA was extracted from cultured cells and tissue samples using Trizol reagent (Life Technologies, Carlsbad, USA). Reverse transcription to cDNA was performed using Prime-Script RT Master Mix (TaKaRa, Dalian, China). Real-time PCR was performed with PrimeSTAR HS DNA polymerase (TaKaRa) on a 7900HT Fast Real-Time PCR System (Applied Biosystems, Foster City, USA). And mRNA expressions were measured by 2-step qRT-PCR using SYBR Green Master Mix kit (Thermo Fisher Scientific, Waltham, USA). *GAPDH* was used as an internal control for normalization of the RNA quantity. The primers used for qRT-PCR are listed in the Supplementary Table S1.

### Lentiviral transduction

MHCC97H cells were seeded in 6-well plates at 5×10 ^5^ cells per well. Then, cells were infected with lentiviral media containing shRNA for SREBP2 (shSREBP2), IL-1β (shIL-1β), or an empty vector when they reached 50% confluence. The stably transfected cell lines were obtained after 1 week of selection using 3 μg/mL puromycin (Life Technologies). The shRNA sequences are listed in the Supplementary Table S2.

### Animal experiments

BALBc nu/nu mice (male, 4 weeks old) were obtained from Shanghai Laboratory Animal Co., Ltd. (Shanghai, China). Subcutaneous implantation models were established by subcutaneously injecting 1×10 ^7^ MHCC97H cells into nude mice. Mice were randomly assigned to 4 groups (five mice per group) after palpable tumors were formed and treated by intragastric administration with PBS (control group), 30 mg/kg lenvatinib, 30 mg/kg betulin, or combination treatment for 3 weeks. Then, tumor samples were extracted for subsequent analysis. All animals received humane care, and all experimental procedures were conducted in conformance with the principles of the National Institutes of Health Guide for the Care and Use of Laboratory Animals (NIH Publications No. 8023) and approved by the Animal Ethics Committee of Fudan University.

### Colony formation assay

For colony formation assays, 300 cells were plated in each well of a 12-well plate and exposed to the treatment for 48 h after attachment. The medium was replaced by fresh medium every three days, and the plates were maintained at 37°C. Fourteen days later, the cell colonies were fixed with 4% paraformaldehyde for 20 min and then stained with 0.1% crystal violet for 15 min. The number of colonies, defined as>50 cells per colony, was counted. Triplicate wells were set up for each condition.

### Cholesterol determination

MHCC97H cells were treated with different concentrations of betulin for 24 h, harvested in RIPA buffer, and extracted with cholesterol/methanol. Total cholesterol level was determined using an Amplex Red Cholesrerol Assay kit (Thermo Fisher Scientific).

### Statistical analysis

Data are presented as the mean±SEM. Depending on the data, Student’s *t* test or one-way ANOVA was performed using GraphPad Prism 7.0 (GraphPad Software) to compare the differences between groups. *P*<0.05 was defined as statistically significant.

## Results

### SREBP2 expression is upregulated in HCC tumor tissues

**Figure 1:**
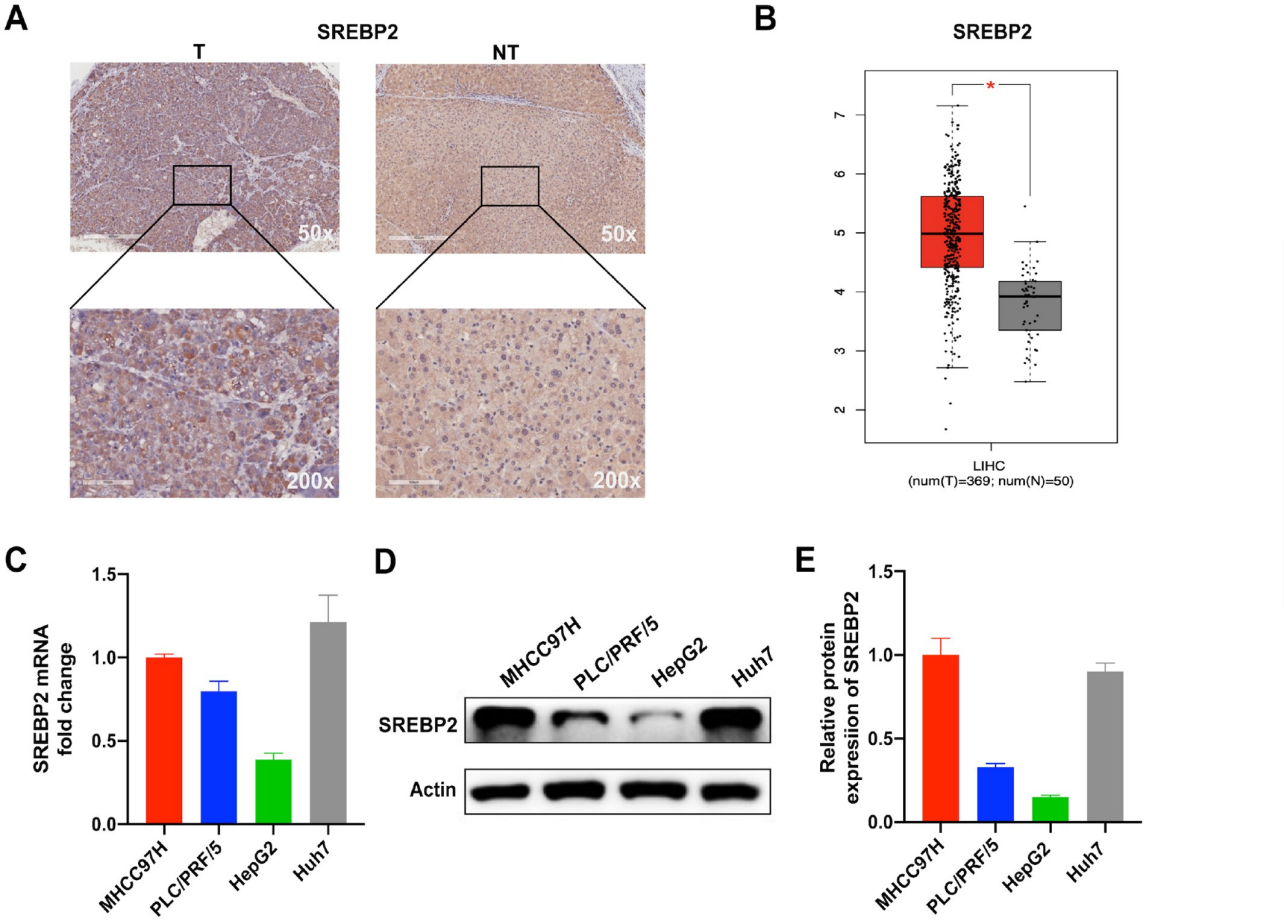
Figure 1 SREBP2 expression is upregulated in HCC tissues (A) Representative IHC image of SREBP2 protein in HCC and adjacent nontumorous tissues. (B) Expression analysis of SREBP2 between tumor and nontumorous tissues in The Cancer Genome Atlas (TCGA) liver hepatocellular carcinoma (LIHC) data. (C) Relative SREBP2 mRNA levels in MHCC97H, PLC/PRF/5, HepG2, and Huh7 cell lines were determined by qRT-PCR. (D,E) Comparison of relative SREBP2 protein expression in four HCC cell lines. *P<0.05.

Inhibition of SREBP2 has been reported to have a broad anti-tumor effect by inhibiting the synthesis of cholesterol [ 16‒ 18]. We applied IHC analysis of an HCC tissue microarray containing 31 paired tumor and nontumor tissues to determine the expression level of SREBP2 in HCC and found that the level of SREBP2 in HCC tumor tissue was higher than that in nontumor tissues ( Figure 1A and Supplementary Figure S1B). Moreover, upregulation of SREBP2 in HCC tumor tissue was also observed in TCGA data ( Figure 1B), and survival analysis indicated that high SREBP2 expression was correlated with poor prognosis in patients with liver cancer ( Supplementary Figure S1A). Then, we detected the expressions of *SREBP2* in MHCC97H, PLC/PRF/5, HepG2, and Huh7 HCC cell lines and found that MHCC97H and Huh7 cells had higher *SREBP2* expression at both the mRNA ( Figure 1C) and protein levels ( Figure 1D,E). Therefore, these two cell lines were used in the subsequent studies.

### SREBP2 inhibitor betulin enhances the antitumour effect of lenvatinib in hepatocellular carcinoma cells

**Figure 2:**
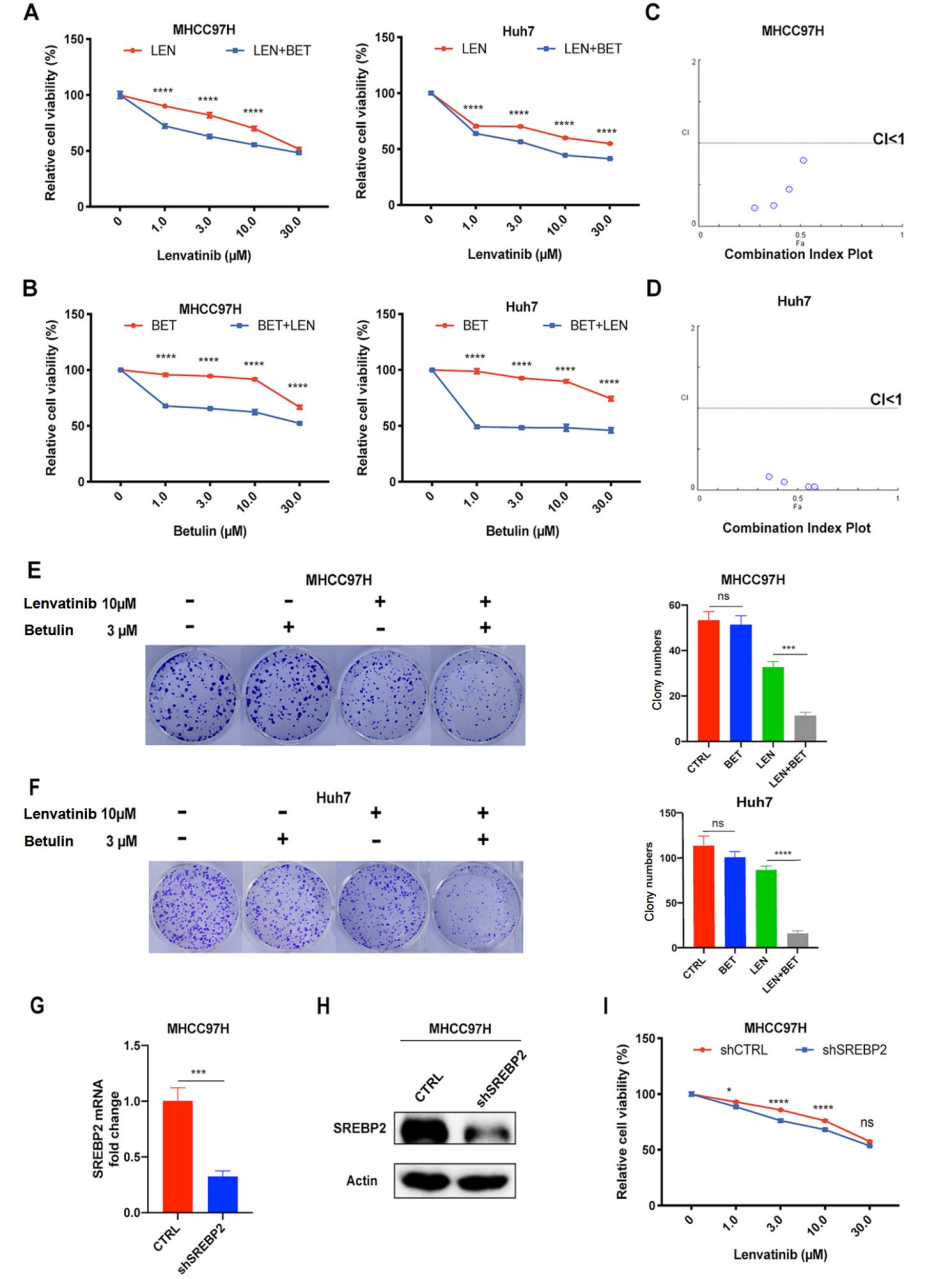
Figure 2 SREBP2 inhibitor betulin enhances the antitumour effect of lenvatinib in HCC cells (A) Effects of different concentrations of lenvatinib with or without betulin (3 μM) on cell viability in MHCC97H and Huh7 cells. (B) Effects of different concentrations of betulin with or without lenvatinib (10 μM) on cell viabilities in MHCC97H and Huh7 cells. (C,D) Combination index (CI) plot of MHCC97H and Huh7 cells treated with lenvatinib (LEN) and betulin (BET) for 72 h. (E,F) Representative images of colony formation assay in MHCC97H and Huh7 cells treated with lenvatinib (10 μM) and betulin (3 μM) alone or in combination and quantification of colony numbers in four groups. (G,H) Western blot analysis and qRT-PCR were used to detect the knockdown efficiency of SREBP2 in MHCC97H cells. (I) Knockdown of SREBP2 enhances the efficacy of lenvatinib in MHCC97H cells. *P<0.05, **P<0.01, ****P<0.0001.

To determine the effect of the SREBP2 inhibitor betulin on the sensitivity of HCC cells to lenvatinib, we first examined the effect of betulin on the cellular cholesterol level. As expected, betulin treatment led to an obvious decrease in the cellular cholesterol content in MHCC97H cells ( Supplementary Figure S1C). Then, the above HCC cells were treated with a combination of lenvatinib and betulin, and we found that betulin enhanced the inhibitory effect of lenvatinib on the growth of Huh7 and MHCC97H cells ( Figure 2A). Similarly, the antiproliferative effect of betulin in HCC cells was also improved by lenvatinib ( Figure 2B). Combination index (CI) analysis was applied to assess the effect of combination treatment [19]. CI=1 indicates an additive effect, CI<1 indicates synergism, and CI>1 represents antagonism. We calculated the CI and found synergism in these two HCC cell lines ( Figure 2C,D). This demonstrates that betulin enhances the antitumour effect of lenvatinib in HCC cells.

To further confirm the enhancement of the SREBP2 inhibitor on lenvatinib’s efficacy, we performed colony formation assay to assess the combined cytotoxic effect of lenvatinib and betulin on HCC cells. We found that combination treatment with lenvatinib and betulin synergistically decreased the clonogenicity of HCC cells ( Figure 2E,F). Then, we performed growth assays in MHCC97H-shCTRL and MHCC97H-shSREBP2 cells. Knockdown efficiency was verified by qRT-PCR and western blot analysis ( Figure 2G,H). The results showed that *SREBP2* knockdown also enhanced the efficacy of lenvatinib ( Figure 2I). Collectively, our results suggest that the combination of betulin and lenvatinib synergistically suppresses HCC cell growth and proliferation *in vitro*.

### Inhibition of IL-1β expression is responsible for the betulin-mediated enhancement of the antitumor effect of lenvatinib

Our previous studies showed that inhibition of SREBP2 suppresses tumor growth and enhances the sensitivity of HCC cells to lenvatinib, but the mechanism is still unclear. Many studies have shown that changes in cellular cholesterol levels are associated with inflammation [ 11, 20 , 21]. Moreover, the increased levels of cytokines may reduce the antitumour effect of TKIs [ 22‒ 24]. Therefore, we explored the expressions of a series of cytokines, including IL-1α, IL-1β, IL-6, and TNF-α, in HCC cells treated with betulin and lenvatinib and found that IL-1β showed the most significant change ( Supplementary Figure S1E).

**Figure 3:**
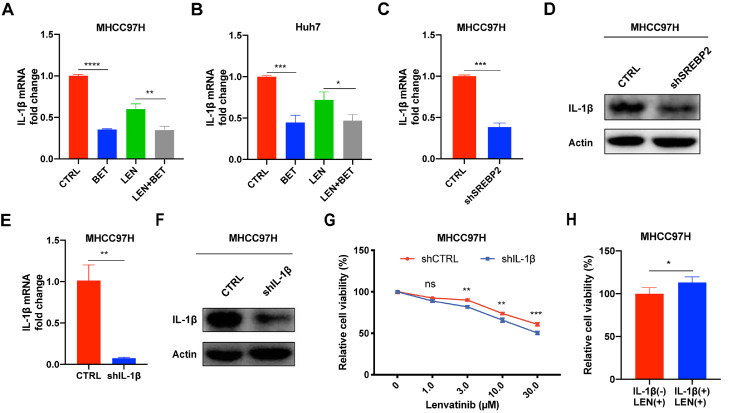
Figure 3 Inhibition of IL-1β expression is responsible for the betulin-mediated enhancement of the antitumour effect of lenvatinib (A,B) Relative intracellular IL-1β levels measured by qRT-PCR in MHCC97H and Huh7 cells. (C,D) Effect of SREBP2 knockdown on intracellular IL-1β level in MHCC97H cells. (E,F) Western blot analysis and qRT-PCR were used to detect the knockdown efficiency of IL-1β in MHCC97H cells. (G) Effect of lenvatinib on the viabilities of MHCC97H-shCTRL and MHCC97H-shIL-1β cells. (H) Effect of exogenous human recombinant IL-1β (20 ng/mL) on the efficacy of lenvatinib in MHCC97H cells. *P<0.05, ** P<0.01, ***P<0.001, ****P <0.0001.

Then, western blot analysis and qRT-PCR were used to detect the expression of IL-1β in MHCC97H-shSREBP2 cells. Compared with those in the MHCC97H-shCTRL cells, the mRNA and protein expression levels of IL-1β were markedly decreased in MHCC97H-shSREBP2 cells ( Figure 3C,D). Next, growth assays were performed in MHCC97H-shCTRL and MHCC97H-shIL-1β cells. Knockdown efficiency was confirmed ( Figure 3E,F), and the results showed that the knockdown of *IL-1β* also enhanced the efficacy of lenvatinib ( Figure 3E,G). Afterward, we detected the effect of exogenous human recombinant IL-1β protein on the efficacy of lenvatinib and found that recombinant IL-1β protein rescued cell viability which was reduced by lenvatinib in MHCC97H cells ( Figure 3H).

### SREBP2 inhibitor enhances the efficacy of lenvatinib by inhibiting the mTOR/IL-1β pathway

**Figure 4:**
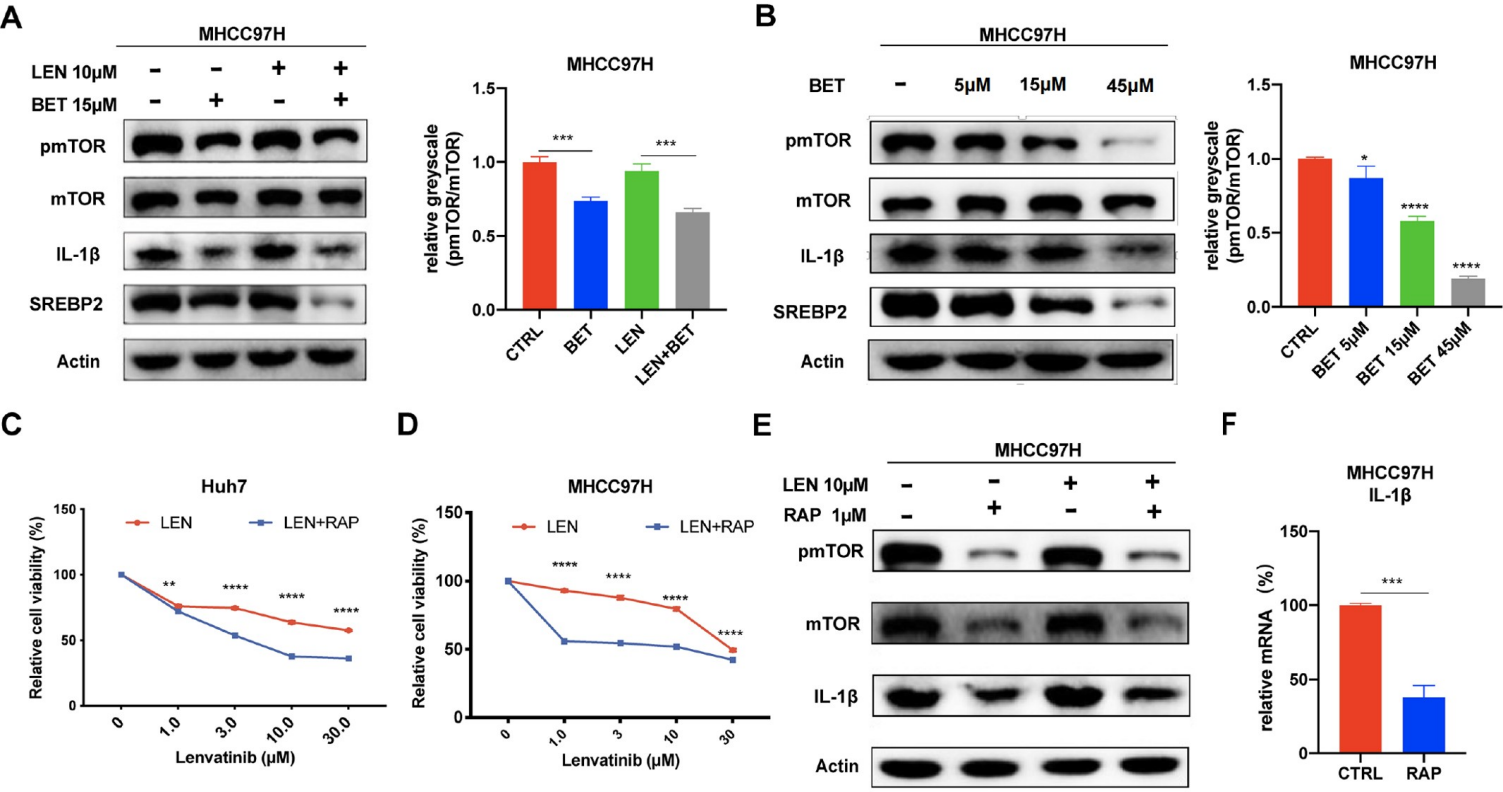
Figure 4 SREBP2 inhibitor enhances the efficacy of lenvatinib by inhibiting the mTOR/IL-1β pathway (A) Western blot analysis of mTOR, pmTOR, IL-1β, and SREBP2 protein levels in MHCC97H cells incubated with lenvatinib (10 μM), betulin (15 μM), or combination treatment. (B) Western blot analysis of mTOR, pmTOR, IL-1β, and SREBP2 protein levels in MHCC97H cells incubated with various concentrations of betulin. (C,D) Effect of different concentrations of lenvatinib with or without rapamycin (1 μM) on cell viabilities in MHCC97H and Huh7 cells. (E) Western blot analysis of mTOR, pmTOR, and IL-1β protein levels in MHCC97H cells incubated with lenvatinib (10 μM), rapamycin (1 μM), or combination treatment. (F) Effect of rapamycin on intracellular IL-1β mRNA level. *P<0.05, ** P<0.01, ***P<0.001, ****P <0.0001.

The mTOR signaling pathway plays a pivotal role in the regulation of cell survival, growth, and proliferation, and this signaling pathway can sense changes in cellular metabolites [ 12, 13, 25 ]. We detected the activation of the mTOR signaling pathway in MHCC97h cells treated with lenvatinib and betulin and found that SREBP2 inhibition suppressed the phosphorylation of mTOR; that is, betulin suppressed the activation of the mTOR signaling pathway alone or with lenvatinib in MHCC97H cells ( Figure 4A). Then, we detected samples treated with different concentrations of betulin and found that the inhibitory effect of betulin on pmTOR, IL-1β, and SREBP2 was dose-dependent ( Figure 4B). To further verify the role of mTOR signaling pathways in regulating the efficacy of lenvatinib, we detected the effect of combination treatment with various concentrations of lenvatinib and the mTOR inhibitor rapamycin on the growth of Huh7 and MHCC97H cells. The results revealed that inhibition of mTOR enhanced the antiproliferative effect of lenvatinib ( Figure 4C,D). Moreover, rapamycin also reduced the expression of IL-1β at both the mRNA and protein levels ( Figure 4E,F). These results demonstrate that the SREBP2 inhibitor enhances the antitumour effect of lenvatinib by inhibiting the mTOR/IL-1β pathway.

### SREBP2 inhibitor enhances the antitumor effect of lenvatinib in a xenograft model

**Figure 5:**
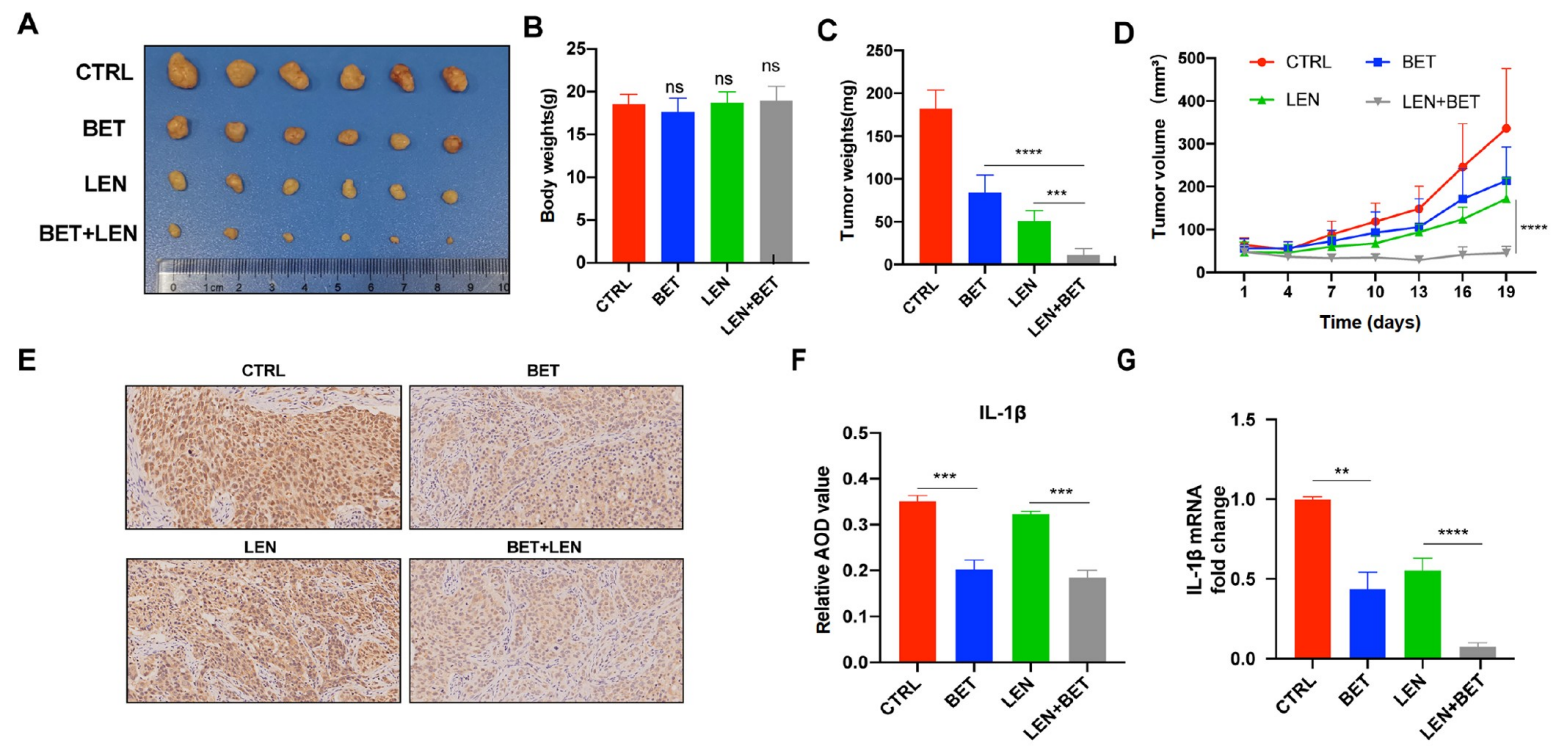
Figure 5 SREBP2 inhibitor enhances the antitumor effect of lenvatinib in the xenograft model Tumor volume (A), body weights (B), and tumor weights (C) among the four groups in the MHCC97H xenograft mouse model at day 19 (n=6). (D) Tumor growth curves in the mouse model after lenvatinib and/or betulin treatment. (E) Representative images of IHC staining for IL-1β from MHCC97H xenografts. (F) Quantitative analysis of the average optical density (AOD) value of IL-1β in each group in (E). (G) IL-1β mRNA level in subcutaneous tumor tissues among the four groups. **P<0.01, ***P<0.001, ****P<0.0001. BET, betulin; LEN, lenvatinib; CTRL, control.

To extend the finding that SREBP inhibition increases the efficacy of lenvatinib, we sought to explore the efficacy of the combination treatment of lenvatinib and betulin on HCC tumor growth *in vivo*. Initially, we established an MHCC97H xenograft model, and then nude mice bearing MHCC97H xenografts were administered with PBS, betulin, lenvatinib, or a combination of betulin and lenvatinib. Growth of the tumors in animals treated with the combination of betulin and lenvatinib was completely arrested, and the size of the tumors was significantly smaller than that in other groups ( Figure 5A,C,D). All treatments were well tolerated in mice, with no significant loss of body weight observed ( Figure 5B). These results were consistent with previous findings *in vitro*.

Next, we validated the mechanisms by which betulin synergizes with lenvatinib in a mouse model. IL-1β expression was detected by IHC and qRT-PCR in tumor tissues of each group. The results showed that betulin alone or with lenvatinib significantly decreased the expression of IL-1β ( Figure 5E‒G), which is consistent with the *in vitro* results ( Figure 3A,B). Overall, our data support the notion that SREBP2 inhibition enhances the antitumor effect of lenvatinib in HCC.

## Discussion

Lenvatinib, a first-line treatment option for advanced HCC, can effectively prolong the survival time of patients. However, the efficacy of lenvatinib is not satisfactory due to side effects and drug resistance [4]. Previous studies have shown that cholesterol metabolism plays an important role in the occurrence and development of tumors and is closely interrelated with many signaling pathways. Moreover, the accumulation of cholesterol in tumor cells is common, especially in HCC [ 16, 26, 27] .

In this study, we found that reducing cellular cholesterol by inhibiting SREBP2 can improve the efficacy of lenvatinib in HCC cells. Sterol regulatory element binding proteins (SREBPs) are a family of transcription factors that regulate cholesterol homeostasis by controlling the expression of a range of enzymes required for endogenous cholesterol, fatty acid, triacylglycerol, and phospholipid synthesis. SREBP2, which belongs to the SREBP family, plays an important role in inhibiting cellular cholesterol synthesis [28]. Our previous work showed that LXR agonists could enhance the antitumor effect of sorafenib in HCC cells by promoting cholesterol efflux [8]. Interestingly, several recent studies have also demonstrated that cellular cholesterol level is associated with the efficacy of TKIs [ 5‒ 7]. These results indicated that lowering cellular cholesterol level increases the sensitivity of tumor cells to TKIs. Thus, whether inhibiting the synthesis of cholesterol has the same effect and which combination regimen is more effective and safer are worthy of further investigation.

In the present study, we found that the SREBP2 inhibitor betulin enhanced the sensitivity of HCC cells to lenvatinib both *in vivo* and *in vitro*. Recently, it was reported that inhibiting the SREBP pathway prevents HCC by downregulating tumor-promoting cytokines, including IL-6, TNF-α, and IL-1β [11]. Therefore, we detected the expressions of a series of cytokines in HCC cells treated with betulin and lenvatinib and found that the decrease in IL-1β was the most dominant, and replenishment of human recombinant IL-1β protein rescued cell viability which was reduced by lenvatinib. Interestingly, some studies also found that elevated levels of some cytokines may reduce the antitumour effect of TKIs [ 22‒ 24]. Our findings confirmed that betulin enhances the sensitivity of HCC cells to lenvatinib by reducing the expression of IL-1β.

Many studies have demonstrated that some signaling pathways, such as the PI3K/AKT/mTOR pathway, RTK/RAS pathway, and SREBP2 pathway, regulate cholesterol synthesis in cancer cells [29]. In turn, mTOR, as a nexus in the growth and proliferation signaling network, can respond to a variety of environmental signals, such as the stimulation of growth factors and changes in metabolites [ 12, 13, 25, 26]. We observed that inhibition of SREBP2 was accompanied by a decrease in mTOR phosphorylation. By combining the mTOR inhibitor rapamycin with lenvatinib, we found that rapamycin not only enhanced the sensitivity of HCC cells to lenvatinib but also reduced the expression of IL-1β. These results further proved that betulin may enhance the sensitivity of HCC cells to lenvatinib by inhibiting the mTOR/IL-1β pathway.

In summary, this study demonstrated that betulin combined with lenvatinib is a promising therapeutic strategy for patients with HCC. To some extent, our study provides a mechanism-based rationale for the action of targeting cholesterol metabolism in cancer treatment. At the same time, there have been many studies exploring the relationship between cholesterol metabolism and the tumor microenvironment [ 30, 31]. The role of cholesterol in the tumor microenvironment and the relationship between the tumor microenvironment and the efficacy of TKIs require further investigation.

## Supporting information

22054Supplementary_figures_and_tables

## Supplementary Data

Supplementary data is available at *Acta Biochimica et Biophysica Sinica* online.
