# Supplementary material for: SREBP2 inhibitor betulin sensitizes hepatocellular carcinoma to lenvatinib by inhibiting the mTOR/IL-1β pathway: SREBP2 Inhibitor sensitizes HCC to lenvatinib
Source: Acta Biochim Biophys Sin (Shanghai). 2023 Jul 11;55(9):1479–86. doi: 10.3724/abbs.2023122 (PMC10520477; doi:10.3724/abbs.2023122)
Supplement: 22054Supplementary_figures_and_tables [file 22054Supplementary_figures_and_tables.pdf]

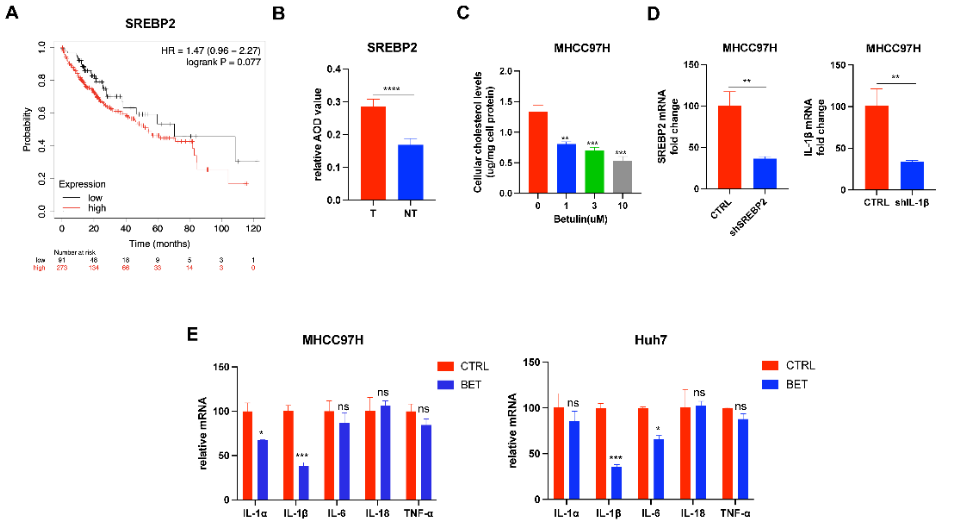

Supplementary Figure S1. Survival analysis of SREBP2 and the effect of Betulin on the level of cholesterol and cytokines in HCC cells (A) Survival probability in patients with liver hepatocellular carcinoma (LIHC) in different expression level of SREBP2 in The Cancer Genome Atlas (TCGA) LIHC data. (B) Quantitative analysis of AOD value of SREBP2 protein in 31 paired HCC and adjacent non-tumorous tissues. (C) The effect of Betulin on total cholesterol level in MHCC97H cells. (D) The knockdown efficiency of SREBP2#2 and IL-1β#2 in MHCC97H cells. (E) The effect of Betulin on the mRNA levels of a series of cytokines in MHCC97H and Huh7 cells.

Supplementary Table S1. Sequences of primers used in this study

Gene	Forward primer (5′→3′)	Reverse primer (5′→3′)
SREBP2	AGCGTCAACAGAGGGACCT	GCAGTGAACCTCCGACTGTATG
IL-1α	TGGTAGTAGCAACCAACGGGA	ACTTTGATTGAGGGCGTCATTC
IL-1β	TTCGACACATGGGATAACGAGG	TTTTTGCTGTGAGTCCCGGAG
IL-18	TCTTCATTGACCAAGGAAATCGG	TCCGGGGTGCATTATCTCTAC
IL-6	CCTGAACCTTCCAAAGATGGC	TTCACCAGGCAAGTCTCCTCA
TNF-α	CCTCTCTCTAATCAGCCCTCTG	GAGGACCTGGGAGTAGATGAG
Actin	GAGCTACGAGCTGCCTGACG	GAGCTACGAGCTGCCTGACG

Supplementary Table S2 Sequences of shRNAs used in this study

shRNA	Sense (5′→3′)	Anti-sense (5′→3′)
shSREBP2#1	CCGGGACCTGAAGATCGAGGACTT TCTCGAGAAAGTCCTCGATCTTCAG GTCTTTTT	AATTAAAAAGACCTGAAGATCGAG GACTTTCTCGAGAAAGTCCTCGATC TTCAGGTC
shSREBP2#2	CCGGCCTCAGATCATCAAGACAGA TCTCGAGATCTGTCTTGATGATCTG AGGTTTTT	AATTAAAAACCTCAGATCATCAAGA CAGATCTCGAGATCTGTCTTGATGA TCTGAGG

shIL-1β#1	CCGGCGGCCAGGATATAACTGACTTCTCGAGAAGTCAGTTATATCCTGGCCGTTTTTG	AATTCAAAAACGGCCAGGATATAACTGACTTCTCGAGAAGTCAGTTATATCCTGGCCG
shIL-1β#2	CCGGCCTGCGTGTTGAAAGATGATACTCGAGTATCATCTTTCAACACGCAGGTTTTTG	AATTCAAAAACCTGCGTGTTGAAAGATGATACTCGAGTATCATCTTTCAACACGCAGG
